# Supplementary material for: Single Ferritin Nanocages Expressing SARS-CoV-2 Spike Variants to Receptor and Antibodies
Source: Vaccines (Basel). 2024 Apr 23;12(5):446. doi: 10.3390/vaccines12050446 (PMC11125617; doi:10.3390/vaccines12050446)
Supplement: Supplementary file 1 [file vaccines-12-00446-s001.zip › vaccines-2943867-supplementary.pdf]

Single ferritin nanocages expressing SARS-CoV-2 Spike variants to receptor and antibodies

Monikaben Padariya[1,*] and Umesh Kalathiya[1,*]

[1]International Centre for Cancer Vaccine Science, University of Gdansk, ul. Kładki 24, 80-822, Gdansk, Poland
*****Correspondence: monikaben.padariya@ug.edu.pl (M.P) and umesh.kalathiya@ug.edu.pl (U.K)

Supporting Materials

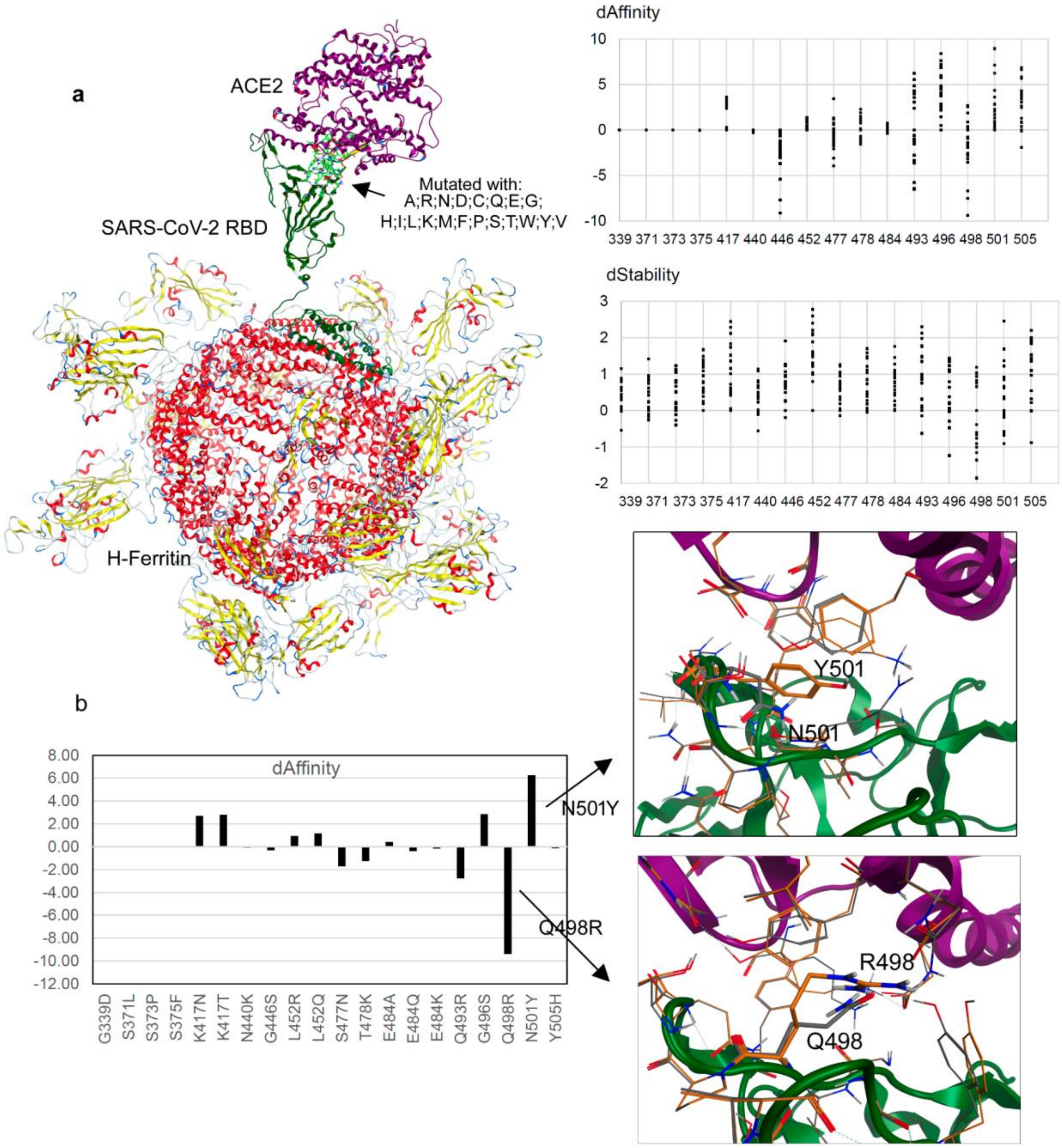

Figure S1. The complex of SARS-CoV-2 Spike RBD-ACE2 was constructed over the H-ferritin nanocage. (a) Mutating residues in the SARS-CoV-2 virus variants (Alpha | B.1.1.7, Beta | B.1.351, Delta | B.1.617.2 & B.1.617, Gamma | P.1, Lambda | C.37, and Omicron | B.1.1.529) were further studied in the presence of different possible mutations (A;R;N;D;C;Q;E;G;H;I;L;K;M;F;P;S;T;W;Y;V) at a particular position using the 'residue scan' approach. The right panel represents the change in the binding affinity and stability (RBD domain) with the ACE2 receptor upon inserting mutations. **(b)** The change in the binding affinity within the Spike RBD-ACE2 complex, upon inserting SARS-CoV-2 virus variants in the RBD domain. The right panel represents the conformational switch of the Q498R and N501Y mutations, describing a contrary behavior.

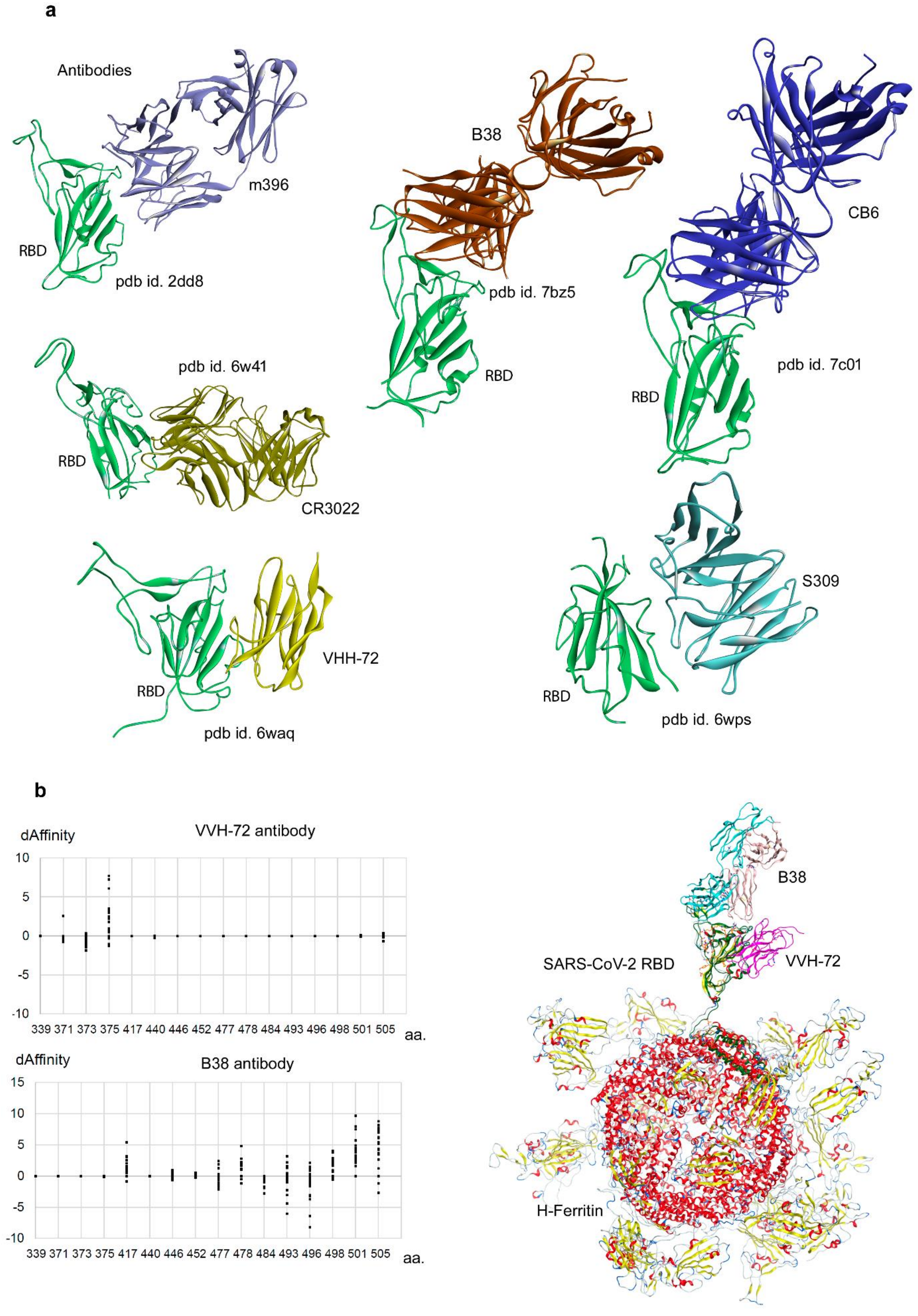

Figure S2. Constructing dataset of mutations in the SARS-CoV-2 Spike RBD structures in the presence of different antibodies. (a) The B38 imitates ACE2 binding to the interface on the RBD of SARS-CoV (pdb. id.: 7bz5), m396 binds to RBD of SARS-CoV without functionally mimicking ACE2, preventing RBD from binding to ACE2 (pdb id.: 2dd8), CB6 functionally mimics ACE2 binding to the interface on RBD of SARS-CoV-2 (pdb id.: 7c01), and

S309 binds to RBD of SARS-CoV-2 without blocking the binding of RBD to ACE2 (pdb id.: 6wps). In addition, the SARS-CoV specific CR3022 binds to RBD of SARS-CoV-2 without blocking the binding of RBD of SARS-CoV-2 to ACE2 (pdb id.: 6w41). **(b)** The change in the binding affinity within the Spike RBD-antibody (VVH-72 or B38) complexes, upon inserting SARS-CoV-2 virus variants in the RBD domain. The right panel represents the VVH-72 or B38 antibody binding with the Spike RBD domain over the H-Ferritin nanocage. The SARS-CoV-2 virus variants (Alpha | B.1.1.7, Beta | B.1.351, Delta | B.1.617.2 & B.1.617, Gamma | P.1, Lambda | C.37, and Omicron | B.1.1.529) were further mutated by different possible mutants (A;R;N;D;C;Q;E;G;H;I;L;K;M;F;P;S;T;W;Y;V) using the 'residue scan' approach.

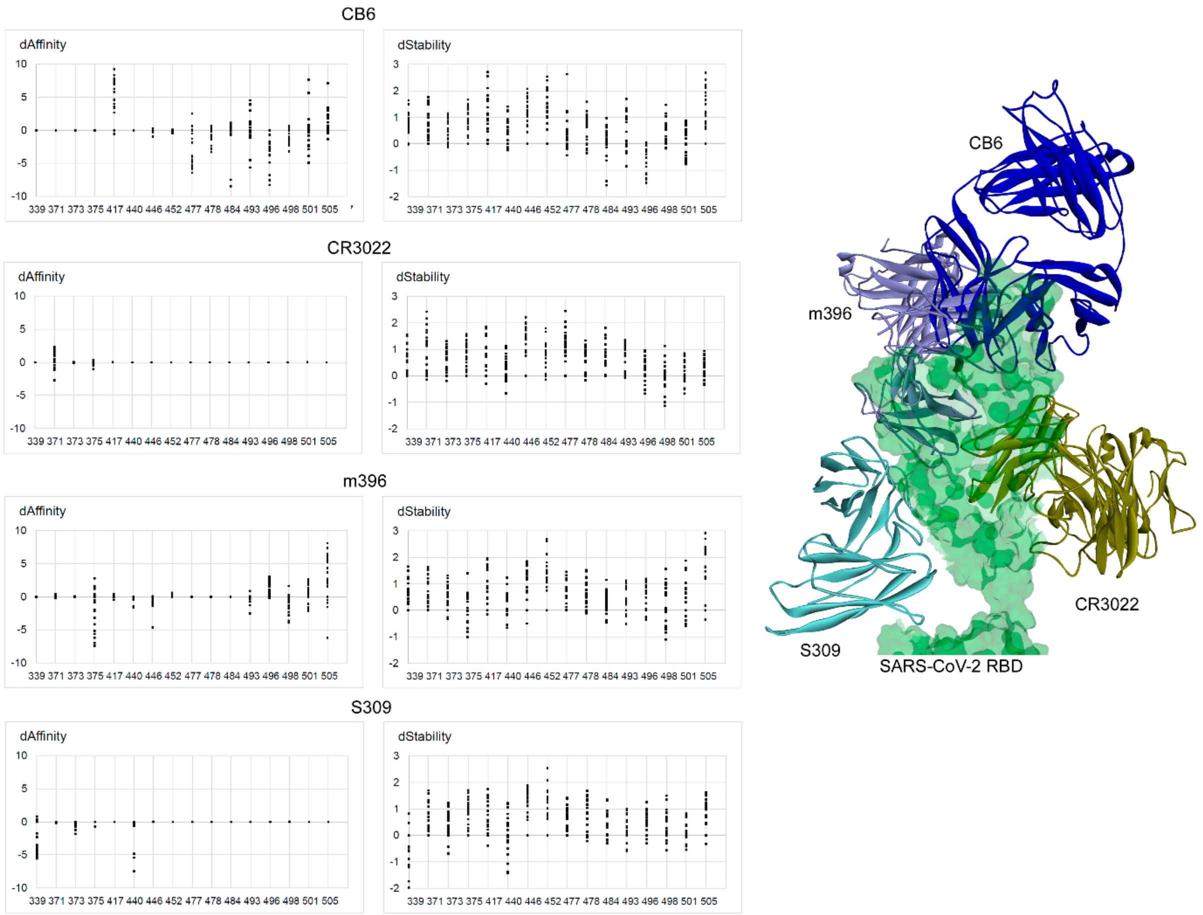

Figure S3. Change in binding affinity with the CB6, CR3066, m396, and S309 antibodies and stability upon inserting point mutations in the SARS-CoV-2 Spike RBD over the H-Ferritin nanocage. The Spike RBD mutated in the presence of m396 (which binds to RBD of SARS-CoV without functionally mimicking ACE2, preventing RBD from binding to ACE2) and CB6 (functionally mimics ACE2 binding to the interface on RBD of SARS-CoV-2), has higher influence over in the binding affinity. The SARS-CoV-2 virus variants (Alpha | B.1.1.7, Beta | B.1.351, Delta | B.1.617.2 & B.1.617, Gamma | P.1, Lambda | C.37, and Omicron | B.1.1.529) were further mutated by different possible mutants (A;R;N;D;C;Q;E;G;H;I;L;K;M;F;P;S;T;W;Y;V) using the 'residue scan' approach. The right panel represents interactions of different antibodies with the Spike RBD domain over the H-ferritin.
